# Prophylactic treatment with bicillin-5 and timalin of convalescence of erysipelas

**DOI:** 10.1186/1471-2334-13-S1-P49

**Published:** 2013-12-16

**Authors:** Liviu Iarovoi, Constantin Andriuță, Raisa Popovici, Stela Cojocaru, Lilia Cojuhari

**Affiliations:** 1 Department of Infectious Diseases, Tropical and Medical Parazitology, Nicolae Testemițanu State Medical and Pharmaceutical University, Chişinău, Republic of Moldova; 2 Toma Ciorbă Clinical Hospital for Infectious Diseases, Chişinău, Republic of Moldova; 3 Department of Infectious Diseases, Faculty for Continuing Medical Education, Nicolae Testemițanu State Medical and Pharmacy University, Chişinău, Republic of Moldova

## Background

Administration of bicillin-5 alone in contemporary conditions of increasing antibiotic resistance and immunodeficiency of population is insufficient to prevent the recurrences of erysipelas. That is why a decision was made to study timalin as an immunomodulatory drug that would prevent along with bicillin-5 the recurrence of erysipelas.

## Methods

52 patients with a recent history of erysipelas and with a high risk of relapse were divided into 2 groups of 26 patients each: one the experimental group in which patients received both bicillin-5 (IM, monthly for 6 or 12 months) and timalin (10 mg IM in a day, in 10 doses) and one control group, in which only bicillin-5 was prescribed. Patients from both groups were supervised epidemiologically, clinically and immunologically for 1-4 years.

## Results

The relapse rate in the experimental group was 2 times lower (19.2%) than in the control group (38.5%). Recurrences of erysipelas in the experimental group occurred during bicillin-5 prophylactic treatment in 2 cases: in the third and sixth month. Out of the 10 patients with relapses in the control group, in 3 the relapses occurred before completing prophylactic treatment with bicillin-5.

## Conclusion

Due to its immunocorrector effects, Timalin lowers the risk of relapses in erysipelas.

